# Evaluating hemoptysis hospitalizations among patients with bronchiectasis in the United States: a population-based cohort study

**DOI:** 10.1186/s12890-021-01762-6

**Published:** 2021-12-01

**Authors:** Rachel K. Lim, Alain Tremblay, Shengjie Lu, Ranjani Somayaji

**Affiliations:** 1 Department of Medicine, University of Calgary, Calgary, Canada; 2 Cumming School of Medicine, University of Calgary, Calgary, Canada; 3 Department of Microbiology, Immunology and Infectious Disease, University of Calgary, Calgary, Canada; 4 Department of Community Health Sciences, University of Calgary, Calgary, Canada

## Abstract

**Background:**

The burden of hospitalizations and mortality for hemoptysis due to bronchiectasis is not well characterized. The primary outcome of our study was to evaluate in-hospital mortality in patients admitted with hemoptysis and bronchiectasis, as well as the rates of bronchial artery embolization, length of stay, and hospitalization costs.

**Methods:**

The authors queried the Nationwide Inpatient Sample (NIS) claims database for hospitalizations between 2016 and 2017 using the ICD-10-CM codes for hemoptysis and bronchiectasis in the United States. Multivariable regression was used to evaluate predictors of in-hospital mortality, embolization, length of stay, and hospital costs.

**Results:**

There were 8240 hospitalizations (weighted) for hemoptysis in the United States from 2016 to 2017. The overall in-hospital mortality was 4.5%, but higher in males compared to females. Predictors of in-hospital mortality included undergoing three or more procedures, age, and congestive heart failure. Bronchial artery embolization (BAE) was utilized during 2.1% of hospitalizations and was more frequently used in those with nontuberculous mycobacteria and aspergillus infections, but not pseudomonal infections. The mean length of stay was 6 days and the median hospitalization cost per patient was USD $9,610. Having comorbidities and procedures was significantly associated with increased length of stay and costs.

**Conclusion:**

Hemoptysis is a frequent indication for hospitalization among the bronchiectasis population. In-hospital death occurred in approximately 4.5% of hospitalizations. The effectiveness of BAE in treating and preventing recurrent hemoptysis from bronchiectasis needs to be explored.

## Introduction

Non-cystic fibrosis bronchiectasis (hereafter referred to as “bronchiectasis”) is a chronic suppurative lung disease characterized by chronic productive cough and episodic infectious exacerbations. Though formerly classified as an orphan disease, its prevalence has steadily increased by ~ 8% per year in the United States (US) [1, 2]. In 2013, an estimated 340, 000 people in the US were receiving active treatments for this disease [3]. Hospitalizations can be frequent and account for most of the economic burden [4]. Numerous clinical trials and guidelines have been published over the past decade and therapies like macrolides are now recommended to prevent bronchiectasis exacerbations [5–10]. There are contraindications to chronic macrolide use though, such as infection by non-tuberculous mycobacteria (NTM) which can develop resistance if macrolide monotherapy is used [11].

Hemoptysis is a well-known and feared complication of bronchiectasis, ranging from chronic small volumes to massive hemoptysis that can be life-threatening. Chronic airway inflammation and infection can lead to hemoptysis but life-threatening hemoptysis may be due to a rupture of a tortuous blood vessel [12]. Bronchiectasis is a common etiology in hemoptysis presentations [1, 13–17]. In a French cohort of bronchiectasis patients, 1 in 5 patients had a history of hemoptysis [18]. Hemoptysis was a symptom in 23% of patients in a US bronchiectasis research registry [19].

Therapeutic approaches to managing bronchiectasis-related hemoptysis depend on the clinical severity but include supportive management such as suspension of certain airway clearance techniques, treatment of pulmonary infection, bronchoscopic techniques, bronchial artery embolization (BAE), and surgical resection. BAE has emerged as a first-line treatment for the control of hemoptysis from a variety of causes, including tuberculosis, bronchiectasis, and aspergilloma [19, 20]. Major complication from BAE are reportedly rare, but the recurrence of hemoptysis is common varying between 10 to 57% [21].

The published literature demonstrates a significant economic burden with bronchiectasis management [1, 2, 22, 23], but there is scarce data on the burden of hospitalizations or mortality rates from hemoptysis or the utilization rates of BAE among the bronchiectasis population in the US. In order to gain a better understanding of the resource and economic impacts of this serious complication, we used a national public database to determine the hospitalization incidence and outcomes of hemoptysis presentations including in-hospital mortality among those with bronchiectasis.

## Methodology

The study cohort was derived from the 2016 and 2017 National Inpatient Sample (NIS) database from the Healthcare Cost and Utilization Project (HCUP). The NIS is a large publicly available all-payer database produced and maintained by the Agency for Healthcare Research and Quality (Rockville, MD) [24]. The NIS provides demographic and administrative data from a 20% stratified random sample of non-federal acute-care hospitals in the US. The study was conducted in accordance with the methodological standards previously published and all investigators had permission to use the data [24]. Given that this study was done using only a publicly available database, ethics approval was not required.

### Variable definitions

The International Classification of Diseases 10^th^ Revision, Clinical Modification (ICD-10-CM) codes were used to identify hospitalizations containing primary or secondary diagnoses of both hemoptysis (R04.2, R04.9) and bronchiectasis (J47.0, J47.1, J47.9). Adults 18 years and older with non-elective admissions were included. Hospitalizations containing ICD-10 codes for bronchogenic malignancy, cystic fibrosis, tuberculosis, iatrogenic hemoptysis related to a procedure, and vasculitis were excluded. Comorbidities were computed with the Elixhauser Comorbidity Software for ICD-10-CM (which includes hypertension, congestive heart failure, renal failure, and diabetes with or without complications) and analysis was adjusted for the number of comorbidities (0, 1–2, ≥ 3). Other data elements from the NIS database were also collected including: age, sex, race, median household income, admission on the weekend, number of procedures, BAE, bronchoscopy, pulmonary mycobacterial infection, pneumonia due to pseudomonas, other pulmonary aspergillosis, Hemophilus influenza infection, sequelae of tuberculosis. Hospitalization costs were calculated by combining charges data with the cost-to-charge ratio files provided by HCUP in order to create standardized costs.

### Statistical analysis

The cohort characteristics were summarized with means or medians for continuous variables and frequencies for categorical variables. The student’s t-test and the Chi-square test were performed to compare differences in continuous and categorical variables, respectively. Baseline variables and outcome measures like the number of hemoptysis-associated hospitalizations were estimated based on the NIS sample, with unweighted results reported directly from the 20% stratified random sample of hospital, and weighted results being estimated by five times the unweighted estimates. The primary outcome was the in-hospital mortality and secondary outcomes included the frequency of BAE, days until BAE, length of stay (LOS), and costs. Socio-demographic and clinical confounders including age, sex, number of procedures by discharge, comorbidities, and income quartile were a priori incorporated into specific multivariable models where appropriate and then finalized using stepwise AIC variable selection. Generalized estimating equations (GEE) were used to construct the multivariable models for mortality, BAE, LOS and costs, and Cox models were constructed for days to BAE. All analyses were conducted with R 3.6.1 (R Core Team, 2019).

## Results

There were a total of 8,240 weighted hospitalizations (1648 unweighted hospitalizations from a 20% stratified random sample of non-federal acute care hospitals in the US) for hemoptysis in persons with bronchiectasis in the study period. The patient demographic and hospital characteristics using the weighted estimates are detailed in Table 1. The mean age was 70 years and females accounted for 58% of the admissions. The majority of patients were Caucasian and had three or more medical comorbidities. Among the weighted hospitalizations, there were estimated 745 with documented *Pseudomonas aeruginosa*, 180 with aspergillus, and 610 with NTM infections. Seventy hospitalizations had a history of prior tuberculosis. The majority of hospitalizations occurred in large centers. There was no notable difference in the incidence of hospitalizations between the two years included in the study. Significant baseline differences between the sexes in this cohort included a higher prevalence of diabetes, renal failure, and congestive heart failure in men.

**Table 1:** Demographics of hemoptysis and bronchiectasis hospitalizations in the United States, 2016–2017

	Males	Females	Total
Number of hospitalizations*	3425	4815	8240
Age in years, mean (standard deviation)	67.8 (15.9)	71.1 (14.7)	69.8 (15.3)
Race, n (%)			
Caucasian	1989 (60.3)	2920 (62.3)	4900 (61.4)
Black	445 (13.5)	565 (12)	1010 (12.7)
Hispanic	390 (11.9)	505 (10.8)	895 (11.2)
Asian/Pacific Islander	305 (9.3)	485 (10.3)	780 (9.9)
Native American	45 (1.4)	20 (0.4)	65 (0.8)
Other	120 (3.7)	195 (4.2)	315 (3.9)
Year, n (%)			
2016	1695 (49.5)	2365 (49.1)	4060 (49.3)
2017	1730 (50.5)	2450 (50.9)	4180 (50.7)
Hospital bed size, n (%)			
Small	445 (13)	705 (14.6)	1150 (14)
Medium	985 (28.8)	1345 (27.9)	2330 (28.3)
Large	1995 (58.2)	2765 (57.4)	4760 (57.8)
Comorbidities, n (%)			
Diabetes	800 (23.3)	865 (17.9)	1665 (20.2)
Renal failure	620 (18.1)	595 (12.4)	1215 (14.7)
Congestive heart failure	765 (22.3)	765 (15.9)	1530 (18.6)
Hypertension	2085 (60.9)	2875 (59.7)	4960 (60.2)
Number of comorbidities, n (%)			
0	155 (4.5)	270 (5.6)	425 (5.2)
1–2	930 (27.2)	1590 (33)	2520 (30.6)
≥ 3	2340 (68.2)	2955 (61.4)	5295 (64.3)

All numbers and covariates are summarized using weighted estimates with exception of mean age

The overall in-hospital mortality was 4.5% and was greater in males (7%) compared to females (3%) (p = 0.01). Hospitalization outcomes by sex are shown in Table 2. Compared to females, males had an adjusted odds ratio for mortality of 1.96 (95% CI 1.15 to 3.33;). Other significant factors associated with increased mortality included age (OR 1.03, 95% CI 1.01–1.05), undergoing three or more procedures during the admission (OR 4.19, 95%CI 1.61–10.92), and having congestive heart failure (OR 2.15, 95% CI 1.19–3.87). Logistic regression models for in-hospital mortality are shown in Table 3.

**Table 2:** Hospital outcomes, comparison by sex

	Males	Females	*P* value
In-hospital deaths	225 (6.6%)	145 (3%)	< 0.0001
No. of bronchoscopy	195 (5.7%)	160 (3.3%)	< 0.0001
No. of BAE	60 (1.8%)	115 (2.4%)	0.0577
Mean LOS (SD)	6.55 (6.67)	5.57 (4.93)	0.0012
Mean hospitalization Costs, $USD (SD)	17, 690 (25, 789)	12, 896 (14, 587)	0.0008

**Table 3:** Odds of in-hospital mortality for persons admitted with hemoptysis

Parameter	Univariate logistic regression	Multivariate logistic regression
	Odds ratio (95% CI)	Odds ratio (95% CI)
Sex		
Male	Ref	Ref
Female	0.44 (0.27, 0.71)	0.51 (0.30, 0.87)
Age		
	1.01 (1.00, 1.03)	1.03 (1.01, 1.05)
Number of procedures		
0	Ref	Ref
1	1.79 (0.77, 4.20)	2.03 (0.88, 4.70)
2	1.43 (0.50, 4.11)	1.55 (0.54, 4.47)
3	3.68 (1.41, 9.56)	4.19 (1.61, 10.92)
4	4.41 (1.61, 12.11)	4.23 (1.52, 11.81)
5	10.01 (3.89, 25.75)	9.78 (3.72, 25.71)
6	6.26 (2.11, 18.56)	6.25 (1.98, 19.73)
7 +	25.74 (11.93, 55.54)	27.29 (12.19, 61.09)
Renal failure		
No	Ref	Ref
Yes	0.90 (0.46, 1.78)	0.46 (0.23, 0.95)
Congestive heart failure		
No	Ref	Ref
Yes	2.50 (1.53, 4.11)	2.15 (1.19, 3.87)
Hypertension		
No	Ref	Ref
Yes	0.81 (0.51, 1.30)	0.61 (0.37, 1.03)

**Fig. 1:**
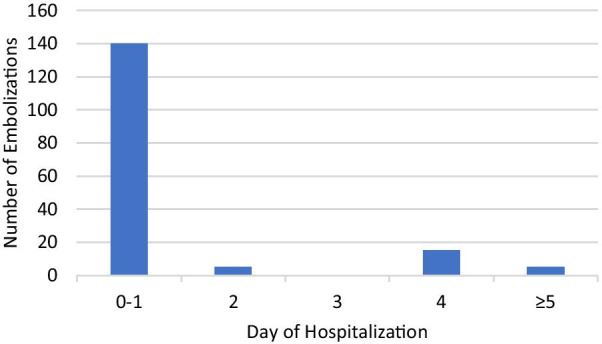
Days to bronchial artery embolization during hospitalization

BAE was performed during 175 (weighted; 2.1%) hospitalizations and 77% took place within the first two days of admission (Fig. 1); bronchoscopy was done in 355 (weighted; 4.3%) of admissions. BAE was repeated during ten hospitalizations. There was no statistically significant difference in BAE utilization or days to BAE between males and females. BAE was more likely to occur in persons with NTM (OR 2.67, 95% CI 1.09 to 6.56; p = 0.03) or aspergillus infection (OR 8.51, 95% CI 3.09 to 23.40; p < 0.001) and in persons who underwent bronchoscopy during the admission (OR 4.93, 95% CI 1.98 to 12.29; p < 0.001); *Hemophilus influenzae* infection was negatively associated with requiring a BAE (p < 0.001). However, no factors were significantly associated with requiring a BAE in a multivariable model. In the Cox model evaluating days to BAE (Table 4), only renal failure was associated with a greater hazard of BAE (aHR 5.11, 95% CI 1.37–19.14).

**Table 4:** Cox model of time to BAE in persons admitted with bronchiectasis-related hemoptysis

Parameter	Univariate	Multivariable
	Hazard ratio (95% CI)	Hazard ratio (95% CI)
Sex		
Male	Ref	Ref
Female	1.20 (0.59, 2.45)	1.41 (0.69, 2.88)
Number of procedures		
1	Ref	Ref
2	0.67 (0.16, 2.80)	0.51 (0.11, 2.29)
3	0.53 (0.20, 1.38)	0.62 (0.20, 1.92)
4	0.89 (0.30, 2.59)	1.18 (0.35, 3.98)
5	2.26 (0.92, 5.56)	3.03 (0.94, 9.75)
6	1.21 (0.39, 3.79)	1.41 (0.37, 5.37)
7 +	0.41 (0.11, 1.53)	0.50 (0.13, 1.86)
Diabetes without chronic complications		
No	Ref	Ref
Yes	2.88 (1.86, 4.44)	3.65 (0.97, 13.72)
Renal failure		
No	Ref	Ref
Yes	2.88 (1.86, 4.44)	5.11 (1.37, 19.14)

The overall mean LOS was 6 days and longer for males (6.55 days) compared to females (5.57 days) (p = 0.001). In the multivariable model undergoing any procedures, having one or more comorbidities, having diabetes (with complications), or having heart failure were significantly associated with greater LOS (Table 5). The overall median hospitalization cost per patient was USD 9,610 (IQR $5,945-$15,846). Costs were significantly lower for females compared to males. In the multivariable model, having any procedures, having one or more comorbidities, and having heart failure had significantly greater associated hospital costs (Table 6).

**Table 5:** Length of stay in persons admitted with bronchiectasis-related hemoptysis

Parameter	Univariate Linear Regression	Multivariate Linear Regression
	Coefficient (95% CI)	Coefficient (95% CI)
Sex		
Male	Ref	Ref
Female	− 0.98 (− 1.56, − 0.39)	− 0.37 (− 0.86, 0.13)
Quartile of income		
1st quartile	Ref	Ref
2nd quartile	− 0.20 (− 0.96, 0.55)	− 0.20 (− 0.85, 0.46)
3rd quartile	− 0.14 (− 0.95, 0.66)	− 0.34 (− 1.05, 0.37)
4th quartile	− 0.41 (− 1.19, 0.38)	− 0.45 (− 1.13, 0.23)
Number of procedures		
0	Ref	Ref
1	0.90 (0.42, 1.38)	0.78 (0.31, 1.24)
2	2.75 (1.90, 3.61)	2.37 (1.51, 3.23)
3	2.64 (1.84, 3.45)	2.34 (1.56, 3.13)
4	2.72 (1.70, 3.74)	2.39 (1.42, 3.36)
5	4.05 (2.65, 5.46)	3.36 (1.93, 4.78)
6	8.80 (5.87, 11.73)	7.33 (4.97, 9.69)
7 +	11.27 (8.36, 14.18)	10.38 (7.53, 13.24)
Number of comorbidities		
0	Ref	Ref
1–2	0.93 (0.29, 1.57)	0.57 (− 0.04, 1.18)
3 +	3.61 (2.93, 4.28)	2.02 (1.38, 2.66)
Diabetes without chronic complications		
No	Ref	Ref
Yes	− 0.93 (− 1.61, − 0.26)	− 1.34 (− 1.99, − 0.68)
Diabetes with chronic complications		
No	Ref	Ref
Yes	2.44 (1.24, 3.64)	0.64 (− 0.48, 1.75)
Congestive heart failure		
No	Ref	Ref
Yes	2.77 (1.89, 3.64)	1.33 (0.53, 2.12)

**Table 6:** Hospitalization costs in persons admitted with bronchiectasis-related hemoptysis

Parameter	Univariate Linear Regression	Multivariate Linear Regression
	Coefficient (95% CI)	Coefficient (95% CI)
Sex		
Male	Ref	Ref
Female	− 4793.44 (− 7584.94, − 2001.94)	− 2340.85 (− 4312.12, − 369.58)
Number of procedures		
0	Ref	Ref
1	3344.03 (2042.37, 4645.70)	3223.95 (1897.39, 4550.51)
2	6596.94 (4816.48, 8377.40)	6279.02 (4461.27, 8096.77)
3	12,812.03 (8496.11, 17,127.95)	12,350.53 (8051.73, 16,649.33)
4	11,231.55 (8307.02, 14,156.09)	10,726.96 (7825.13, 13,628.79)
5	14,661.62 (9100.61, 20,222.63)	13,452.02 (7949.11, 18,954.93)
6	30,944.28 (19,663.22, 42,225.33)	29,557.91 (18,505.43, 40,610.40)
7 +	57,907.32 (39,326.48, 76,488.17)	55,684.24 (37,486.24, 73,882.25)
Number of comorbidities		
0	Ref	Ref
1–2	3362.38 (1112.09, 5612.67)	2865.29 (632.83, 5097.75)
3 +	9697.50 (7447.33, 11,947.67)	4759.30 (2120.37, 7398.24)
Congestive heart failure		
No	Ref	Ref
Yes	8941.26 (4059.18, 13,823.33)	4611.69 (1113.74, 8109.64)
Hypertension		
No	Ref	Ref
Yes	− 788.90 (− 3561.97, 1984.18)	− 2526.54 (− 4987.84, − 65.24)

## Discussion

In our large study evaluating the burden of bronchiectasis-related hemoptysis admissions across 20% of acute-care hospitals in the United States, there were 1648 admissions (unweighted) for hemoptysis and bronchiectasis between 2016 & 2017. In comparison, a prior study using the same database reported 9746 admissions (unweighted) for hemoptysis and bronchiectasis between 1993 and 2006 (approximately 750 hospitalizations per year) [1]. It remains unclear whether the frequency of hospitalizations for bronchiectasis-related hemoptysis is increasing based on our results. However, with an increasingly aging population, the prevalence of bronchiectasis and its complications are expected to continue rising. Given that bronchiectasis is typically an incurable and chronic condition, the underlying inflammation and destruction of lung can lead to recurrent bleeding and hospitalizations.

Small studies have demonstrated that bronchiectasis exacerbations carry an in-hospital mortality rate ranging from 9–35% [25–27]. Our study showed a lower proportion of in-hospital death of 4%. The risk of death was significantly higher among males, which is similar to results by Finklea and colleagues [25]. In this cohort, males had a higher prevalence of diabetes, renal failure, and congestive heart failure compared to females. Not surprisingly, these specific diseases as well as multi-morbidity have a negative impact on survival, as demonstrated in an international cohort of 986 patients with bronchiectasis [28].

Our analysis showed that BAE was performed in 2.1% of admissions for hemoptysis related to bronchiectasis. The relatively low rate of BAE utilization may suggest that there was a low frequency of life-threatening hemoptysis, although other potential explanations include delayed presentations, variation in health resources and clinical practice, and lack of health insurance coverage. While BAE is considered first-line therapy for massive hemoptysis, the role of BAE in non-massive hemoptysis is less clear. A study demonstrated that moderate hemoptysis carried a poor prognosis with a mortality rate of 4–12% similar to those with massive hemoptysis [29]. Recurrent hemoptysis was significantly lower in those who underwent BAE (30%) compared to those without BAE (91%) during a median follow-up time of 2.5 years [29]. Recurrent hemoptysis was also less frequent if BAE was done within 24 h of admission. In our analysis, 82% of BAE procedures were conducted within the first 48 h of admission and were repeated during only ten admissions. Further studies are needed to see if BAE can reduce hospitalizations and prevent readmissions in bronchiectasis patients with non-massive hemoptysis.

BAE was more frequently used in patients with NTM or aspergillus infections although there was a very small proportion with either of these infections in the cohort to enable detailed analyses. Hemoptysis is commonly experienced in those with NTM lung disease, and there are successful reports of BAE in the literature [30, 31]. Aspergillus-associated hemoptysis is typically related to aspergillomas, as well as chronic pulmonary aspergillosis [32]. Whether the acute management of hemoptysis should differ in those with these chronic infections compared to others with bronchiectasis is unclear, however, antimicrobials and surgery can also be considered in cases where the infection is deemed causative. Renal failure was associated with longer days until BAE, which may stem from a fear of contrast-induced nephropathy with renal dysfunction [33].

There are a number of limitations to this study based solely on administrative data. Coding errors can lead to underreported or misclassified diagnoses. Claims data also do not contain clinical details on an individual level, such as complete medical comorbidities, medications, or history of prior hemoptysis or exacerbations. This lack of detail limits the analysis, such as stratification by the quantity of hemoptysis or severity of underlying bronchiectasis and prevents the authors from focusing on certain groups like those with life-threatening hemoptysis. While other common causes of hemoptysis like tuberculosis, cystic fibrosis, vasculitis, and lung malignancy were excluded, the authors cannot confirm that hemoptysis was directly caused by bronchiectasis or bronchiectasis exacerbations and therefore, rarer causes of hemoptysis may have been included in the analysis. Finally, the NIS reports data from hospitalizations, not individual patients, and readmissions may have been included in this retrospective cohort. Regardless, the absolute number of admissions for this complication is an important finding to understanding the burden of the disease. This is also one of the largest studies to examine bronchiectasis-related hemoptysis hospitalizations on a population level.

## Conclusion

Hemoptysis in this patient cohort was associated with significant mortality and morbidity with some sex-based differences found in LOS, hospitalization costs, and in-hospital mortality. Health care systems should adjust resources accordingly to accommodate this cost and further research into the role of bronchial artery embolization in non-massive hemoptysis is needed.

## Data Availability

The data that support the findings of this study are available from the NIS database but restrictions apply to the availability of these data, which were used under license for the current study, and so are not publicly available. Data are however available from the authors upon reasonable request and with permission of HCUP. (https://www.hcup-us.ahrq.gov/**).**
